# Biomarkerdiagnostik bei Karzinomen des oberen Gastrointestinaltrakts

**DOI:** 10.1007/s00292-025-01506-x

**Published:** 2025-12-10

**Authors:** Drolaiz H. W. Liu, Gudrun Piringer, Sabina Köfler, Rupert Langer

**Affiliations:** 1 Klinisches Institut für Pathologie und Molekularpathologie, Johannes Kepler Universität Linz und Kepler Universitätsklinikum GmbH, Krankenhausstr. 5–8, 4021 Linz, Österreich; 2 Department of Pathology, GROW School for Oncology and Reproduction, Maastricht University Medical Center, Maastricht, Niederlande; 3 Klinik für Hämatologie und Internistische Onkologie, Johannes Kepler Universität Linz und Kepler Universitätsklinikum GmbH, Linz, Österreich

**Keywords:** Ösophaguskarzinom, Magenkarzinom, HER2, PD-L1, Mikrosatelliteninstabilität, Claudin 18.2, Esophageal cancer, Gastric cancer, HER2, PD-L1, Microsatellite instability, Claudin 18.2

## Abstract

Die prädiktive Biomarkertestung ist mittlerweile integraler Bestandteil der modernen Diagnostik und Therapieplanung bei Ösophagus- und Magenkarzinomen. Sie besteht aktuell überwiegend aus immunhistochemisch bestimmbaren Markern, wie der Expression des „programmed death ligand 1“ (PD-L1) bei (ösophagealen) Plattenepithelkarzinomen (angegeben mittels Tumor Proportion Score [TPS], Combined Positivity Score [CPS] und Tumor Area Proportion [TAP] Score), der HER2-Status (ergänzt durch In-situ-Hybridisierung bei nichteindeutigen Ergebnissen), der Mismatch-repair-Status (fakultativ ergänzt durch die molekularpathologische Bestimmung des Mikrosatellitenstatus) und seit Mitte des Jahrs 2024 auch die Expression von Claudin 18.2 bei gastroösophagealen Adenokarzinomen. Eine Erweiterung des Panels durch FGFR2b ist in naher Zukunft zu erwarten. Neben der den Qualitätsrichtlinen entsprechenden technischen Durchführung der Untersuchungen ist auch die biologische Heterogenität vieler dieser Biomarker eine diagnostische und klinische Herausforderung. Dieser Artikel gibt einen Überblick über die aktuelle Biomarkerdiagnostik bei Tumoren des oberen Gastrointestinaltrakts und setzt diese in einen entsprechenden onkologisch-therapeutischen Kontext.

## Abstract (en)

Predictive biomarker testing is nowadays an integral part of modern diagnostics and treatment planning for esophageal and gastric cancer. It currently consists mainly of immunohistochemically determined markers, namely the PD-L1 status (stated by the Tumor Proportion Score [TPS], Combined Positivity Score [CPS] and Tumor Area Proportion [TAP] Score) in esophageal squamous cell carcinomas, as well as the HER2 status (supplemented by in situ hybridization in case of equivocal results), the mismatch repair status (optionally supplemented by molecular pathological determination of microsatellite status), and, since mid-2024, the expression of claudin 18.2 in gastroesophageal adenocarcinomas. An expansion of the panel by FGFR2b is expected in the near future. In addition to the quality guideline-compliant technical performance of the tests, the biological heterogeneity of many of these biomarkers is also a diagnostic and clinical challenge. The current article provides an overview of current biomarker testing in the context of current treatment options for cancer of the upper gastrointestinal tract.

## Lernziele

Nach Lektüre dieses Beitrags …

- können Sie die obligat zu testenden Biomarker für die zugelassenen zielgerichteten Therapien bei diesen Tumoren benennen;
- kennen Sie die klinischen Konsequenzen eines Testergebnisses;
- sind Ihnen die Herausforderungen der Biomarkerdiagnostik im Hinblick auf technische und biologische Besonderheiten bekannt;
- wissen Sie, weitere potenzielle Biomarker einzuordnen;
- sind Sie in der Lagen, die klinisch tätigen Kollegen auf Basis der molekularpathologischen Befunde bezüglich der weiteren möglichen Therapieoptionen zu beraten.

## Einleitung

Bei **HER2-positiven Adenokarzinomen**

HER2-positiven Adenokarzinomen

des Magens und des gastroösophagealen Übergangs (GEJ) im lokal fortgeschrittenen, inoperablen oder metastasierten Stadium zeigte die ToGA-Studie im Jahr 2010 erstmals einen Überlebensvorteil und eine höhere Ansprechrate für die Therapie mit Trastuzumab in Kombination mit Chemotherapie (Cisplatin plus Capecitabin oder 5‑Fluorouracil [5-FU]; [1]). Das HER2-Protein stellt damit die am längsten etablierte **molekulare Zielstruktur**

molekulare Zielstruktur

beim Magenkarzinom/GEJ dar. Die Bestimmung des HER2-Status war im Anschluss daran für weitere Jahre die einzige therapeutisch relevante Biomarkerdiagnostik bei diesen Tumoren. Die Ergebnisse rezent publizierter und vielbeachteter klinischer Studien haben jedoch mittlerweile zu einem Paradigmenwechsel bei den **zielgerichteten Therapien**

zielgerichteten Therapien

für Ösophagus‑, Magen- und gastroösophageale Übergangskarzinome geführt und zwar nicht nur im metastasierten Stadium, sondern auch in der perioperativen Situation bei operablen Tumoren: Therapien mit **Checkpointinhibitoren**

Checkpointinhibitoren

(z. B. Pembrolizumab, Nivolumab, oder Tislelizumab; [2, 3, 4]) sowie seit Mitte des Jahrs 2024 die gegen das Tight-junction-Protein Claudin (CLDN) 18.2 gerichtete Therapie mit Zolbetuximab werden zunehmend eingesetzt [5].

Der nachfolgende Überblick stellt die aktuelle Biomarkerdiagnostik bei Ösophagus- und Magenkarzinomen in den Kontext der klinisch-onkologischen Therapieoptionen.

## Therapeutisch relevante Biomarker

Die Biomarkerdiagnostik umfasst aktuell im Wesentlichen die Bestimmung des Status von „programmed death ligand 1“ (PD-L1) bei ösophagealen Plattenepithelkarzinomen sowie bei gastroösophagealen Adenokarzinomen die Bestimmung des HER2-, Mikrosatelliten- bzw. Mismatch-repair(MMR)- und PD-L1-Status sowie seit Mitte 2024 auch die Bestimmung der Expression von CLDN18.2 (Tab. 1). Nach präliminären Studienergebnissen ist zudem zu erwarten, dass dieses Panel bald um die Bestimmung der 2b-Isoform des Fibroblastenwachstumsfaktorrezeptors 2 (FGFR2b) erweitert wird [6, 7].

**Tab. 1:** Derzeit empfohlene Biomarker für Karzinome des oberen Gastrointestinaltraktes

Marker	Methode	Bemerkung
HER2	IHC/ISH	Adenokarzinom – Beurteilung der Intensität von 0 bis 3+; bei grenzwertigen Befunden (2+) ergänzend FISH/SISH/CISH; unterschiedliche Kriterien für Biopsien und Resektate hinsichtlich der Menge der als positiv gewerteter Tumorzellen für die Gesamtbewertung
MMR/MSI	IHC/Molekularpathologisch	Adenokarzinom – meist IHC, alternativ PCR oder NGS; Übereinstimmung zwischen IHC und PCR bei über 98 %
PD-L1	IHC	TPS (%) und CPS (numerisch) für Plattenepithelkarzinom und Adenokarzinom TAP Score (%) nahezu gleichwertig mit CPS, aber leichter zu bestimmen
CLDN18.2	IHC	Adenokarzinom – Positivität definiert als ≥ 75 % der Tumorzellen mit membranöser Färbung von 2+ oder 3+, analog zu HER2; Zweitbegutachtung bei Werten von 60–80 % empfohlen

*CISH* chromogene ISH,* CPS *Combined Positive Score,* IHC* Immunhistochemie, *ISH* In-situ-Hybridisierung, *MMR* „mismatch repair“, *MSI* Mikrosatelliteninstabilität, *NGS* Next Generation Sequencing, *PCR* Polymerasekettenreaktion, *PD-L1* „programmed death ligand 1“, *SISH* Silber-ISH, *TAP* Tumor Area Positivity, *TPS* Tumor-proportion-Score

### Merke

Die Biomarkerdiagnostik für Karzinome des Oberen Gastrointestinaltraktes erfolgt überwiegend mittels Immunhistochemie, ergänzt durch In-situ Hybridierung.

Die Expression einiger dieser Biomarker zeigt eine enge Assoziation zu molekular definierten Subgruppen, insbesondere des Magenkarzinoms (nach The Cancer Genome Atlas [TCGA]: Ebstein-Barr-Virus [EBV], MSI, chromosomale Instabilität [CIN], genomisch stabil [GS]), die wiederum eine Assoziation mit den morphologischen WHO- bzw. Lauren-Subtypen aufweisen. Die **CIN-Subgruppe**

CIN-Subgruppe

ist am häufigsten und zeichnet sich durch Amplifikationen von Rezeptortyrosinkinasegenen (u. a. HER2, FGFR2) aus. Die EBV-positiven Tumoren zeigen eine hohe Immunogenität und PD-L1-Expression. Die MSI-Tumoren entstehen durch Defekte im MMR-System, meist infolge von MLH1-Methylierung. Die GS-Tumoren sind oft vom wenig kohäsiven Typ, betreffen jüngere PatientInnen und weisen Mutationen in CDH1 oder RHOA auf [8]. Diese molekulare Vielfalt spiegelt sich auch in der Heterogenität der Biomarkerexpression wider und beeinflusst den Behandlungsentscheid im Hinblick auf eine individualisierte Therapie.

### Merke

Die therapierelevanten Marker für Adenokarzinome des oberen Gastrointestinaltraktes sind der Mikrosatelliten-Status, Her2, PD-L1 und Claudin (CLDN) 18.2.

### Merke

Beim Plattenepithelkarzinom des Ösophagus ist derzeit nur PD-L1 als therapierelevanter Marker relevant.

### HER2

Das Gen* ERBB2* (Alias: *HER2*) kodiert für HER2, einen Tyrosinkinaserezeptor der ErbB-Rezeptor-Familie, dessen Überexpression und/oder Genamplifikation zur Aktivierung proliferationsfördernder und antiapoptotischer Signalwege führt. In gastroösophagealen Adenokarzinomen findet sich eine **HER2-Überexpression**

HER2-Überexpression

in 8–20 % der Fälle, wobei eine Häufung bei intestinalen/tubulären Karzinomen im distalen Ösophagus, am gastroösophagealen Übergang und im proximalen Magen zu beobachten ist [8].

Die Bestimmung des HER2-Status erfolgt primär mittels **Immunhistochemie**

Immunhistochemie

(IHC). Im Unterschied zum Mammakarzinom zeigen Adenokarzinome des oberen Gastrointestinaltrakts häufiger eine unvollständige (basolaterale/laterale) Membranexpression, was ebenfalls als positive Reaktion zu werten ist. Für die Auswertung hat sich die **„Magnifikationsregel“**

„Magnifikationsregel“

bewährt [9]: Eine Intensität von 3+ entspricht einer kräftigen braunen „chicken-wire“-artigen Immunreaktivität, die bereits bei 50-facher Vergrößerung sichtbar ist. Für Färbungen der Intensität 2+ ist eine 100- bis 200-fache Vergrößerung erforderlich, 1+ wird erst bei 400-facher Vergrößerung sichtbar. Bei Resektaten gilt eine Positivität ab ≥ 10 % der Tumorzellen mit starker membranöser Expression (Score 3+), bei Biopsien bereits ab 5 zusammenliegenden Tumorzellen mit starker Expression. Für äquivoke IHC-Befunde (2+) ist die **In-situ Hybridisierung**

In-situ Hybridisierung

(ISH; Fluoreszenz-ISH [FISH], Silber-ISH [SISH] oder chromogene ISH [CISH]) zur Bestimmung der ***HER2*-Gen-Amplifikation**

**Abb. 1:**
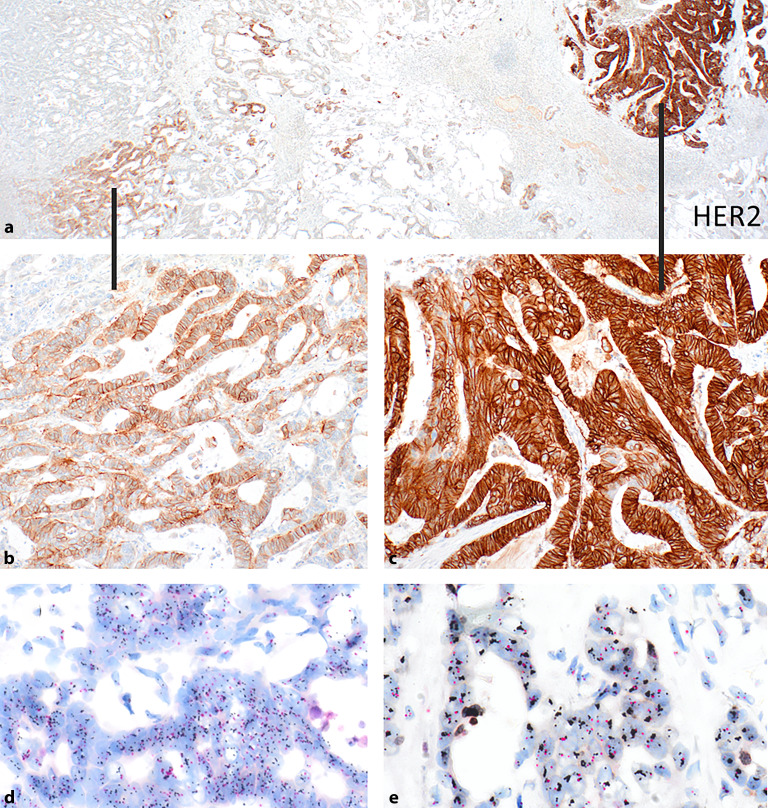
Beispiel einer heterogenen immunhistochemischen Expression von HER2 in einer Metastase eines Adenokarzinoms des Magens (tubulärer/intestinaler Typ). **a** Übersicht: rechts ein Areal mit einem Expressionslevel von 3+ (Detail in **c**), links ein Areal mit einem Expressionslevel von 2+ (Detail in **b**); dazwischen HER2-negative Abschnitte. **d** *HER2*-Amplifikation dargestellt in einer chromogenen In-situ-Hybridisierung (CISH; aus Areal **c**). **e** High-level-*HER2*-Amplifikation mit Cluster (aus Areal **d**)

HER2-Gen-Amplifikation

obligat. Die Auswertung erfolgt anhand des Quotienten aus* HER2 *und einer Zentromerprobe für Chromosom-17 (CEP17): Ein Quotient ≥ 2 oder > 6 *HER2*-Kopien pro Zelle zeigt eine Amplifikation an und der Tumor wird als HER2-positiv gewertet. Quotienten < 2 und <4 Kopien werden als negativ gewertet, Werte dazwischen als äquivok, diese bedürfen der weiteren Analyse z. B. durch eine andere ISH-Technik ggf. in einem anderen Labor ([9]; Abb. 1).

Der HER2-Status ist für die Auswahl der Erstlinientherapie beim fortgeschrittenen oder metastasierten Magenkarzinom entscheidend. Bei HER2-positiven Tumoren stellt Trastuzumab in Kombination mit Chemotherapie die **Standardbehandlung**

Standardbehandlung

da. In der Zweitlinie steht mit Trastuzumab-Deruxtecan eine neue Option zur Verfügung, wobei eine erneute HER2-Testung empfohlen wird. Kombinationen von Trastuzumab mit Immuntherapien (z. B. Pembrolizumab) zeigen in aktuellen Studien einen zusätzlichen Überlebensvorteil, insbesondere bei PD-L1-positiven Tumoren [10].

### PD-L1

Das **Immuncheckpointprotein**

Immuncheckpointprotein

PD-L1 wird von Tumorzellen und tumorassoziierten Immunzellen exprimiert. Über die Bindung an den PD-1-Rezeptor auf T‑Zellen hemmt es die Immunantwort und ermöglicht es den Tumorzellen, der Immunüberwachung zu entgehen. Die therapeutische Blockade dieser Achse mit monoklonalen Antikörpern (z. B. Pembrolizumab, Nivolumab, Tislelizumab) hat die Behandlungsergebnisse bei Magenkarzinomen, GEJ-Adenokarzinomen und Plattenepithelkarzinomen des Ösophagus signifikant verbessert, insbesondere bei hoher PD-L1-Expression [2, 4, 11].

Die Bestimmung der PD-L1-Expression erfolgt immunhistochemisch. Eine Immunreaktivität wird sowohl in Tumorzellen beobachtet – hier jedoch deutlich weniger als z. B. bei Lungenkarzinomen – als auch in Immunzellen, inklusive Lymphozyten, Makrophagen und neutrophilen Granulozyten [12]. Für die **PD-L1-Testung**

**Abb. 2:**
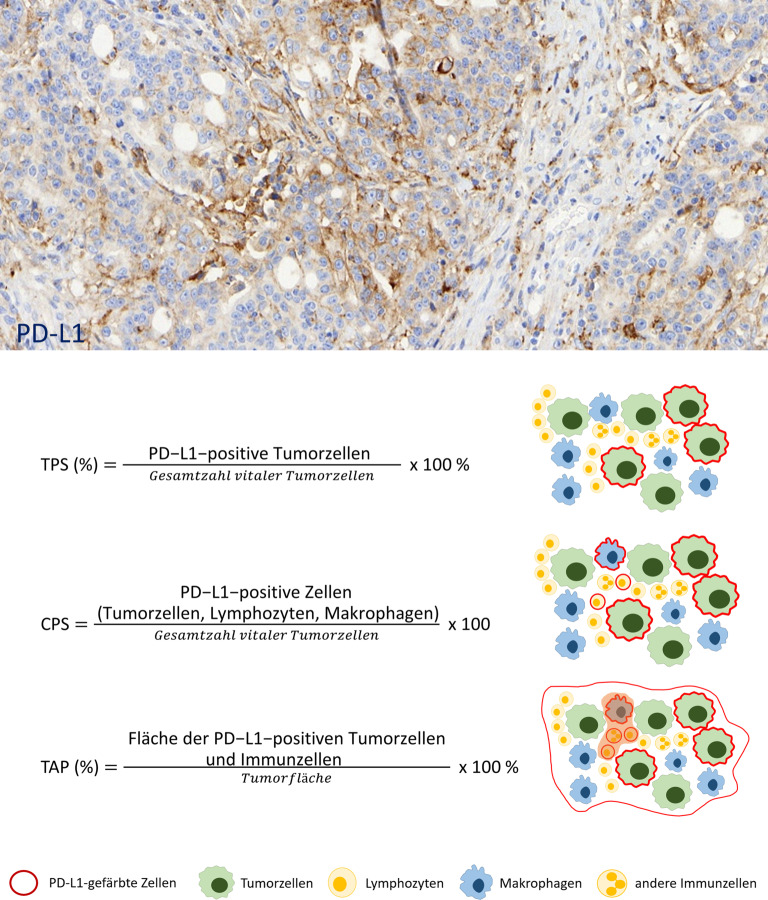
Beispiel einer Färbung von „programmed death ligand 1“ (*PD-L1*) mit positiver Reaktion in Tumorzellen und Immunzellen sowie Überblick über die 3 PD-L1-Scoring-Systeme Tumor Positivity Score (*TPS*), Combined Positivity Score (*CPS*) und Tumor Area Positivity (*TAP*) Score

PD-L1-Testung

stehen verschiedene kommerzielle Assays zur Verfügung, die weitgehend austauschbar sind [13]. Für das Scoring der PD-L1-Expression wurden im Rahmen der Zulassungsstudien mehrere Auswertungssysteme angewandt [12], die im Folgenden beschrieben werden (Abb. 2).

#### Combined Positive Score

Der Combined Positive Score (CPS) ist das am weitesten verbreitete Bewertungssystem für die PD-L1-Bestimmung in GEJ-Karzinomen. Er berücksichtigt sowohl PD-L1-positive Tumorzellen als auch mononukleäre Immunzellen (Lymphozyten, Makrophagen) im Verhältnis zur Gesamtzahl vitaler Tumorzellen. Der CPS wird als absoluter Wert (0–100) angegeben. Die Begriffe „positiv“ oder „negativ“ sollten vermieden werden, da verschiedene Therapien **unterschiedliche Cut-off-Werte**

unterschiedliche Cut-off-Werte

nutzen. Die Bewertung erfolgt ausschließlich im invasiven Tumoranteil, wobei Nekrosen, Ulzera und nichttumoröse Areale ausgeschlossen werden. Tumorzellen mit jeglicher membranöser Färbung gelten als positiv; auch Immunzellen im direkten Tumorumfeld werden berücksichtigt. Die praktische Umsetzung des CPS erfordert eine **detaillierte Auszählung**

detaillierte Auszählung

aller relevanten Zellen im Tumorareal, was an immunhistochemisch gefärbten Schnitten mit dicht liegenden, sich überlappenden Zellen und mit oft nur Hämatoxylin als Gegenfärbung schwierig sein kann. Besonders herausfordernd ist die Unterscheidung von Lymphozyten und Makrophagen gegenüber anderen Zellen, wie Plasmazellen, Neutrophilen oder Fibroblasten, die nicht in die CPS-Berechnung einfließen.

#### Tumor Area Positivity Score

Der Tumor Area Positivity (TAP-)Score ist ein neueres Bewertungssystem, das initial für **Tislelizumab**

Tislelizumab

bei ösophagealen Plattenepithelkarzinomen und Übergangskarzinomen etabliert wurde, mittlerweile aber auch im Studiensetting für andere Checkpointinhibitoren Verwendung findet [14]. Hier wird der Anteil der gesamten Tumorfläche (Tumorzellen und tumorassoziierte Immunzellen mit membranöser Färbung innerhalb des desmoplastischen Stromas), der eine PD-L1-Expression zeigt, visuell geschätzt. Als klinisch relevant für Tislelizumab gilt derzeit ein TAP-Score ≥ 5 %. Der TAP-Score korreliert eng mit dem CPS, ist aber aufgrund des Konzepts der Flächenbestimmung und der Einbeziehung aller Immunzellen, inklusive z. B. neutrophiler Granulozyten, einfacher in der Handhabung, zeigt eine etwas bessere Interobserver-Übereinstimmung und ist für bei Verwendung eine **digitalen Bildauswertung**

digitalen Bildauswertung

besser geeignet. Eine Ausweitung der Anwendung des TAP-Scores auf die Indikationsstellung für andere Immuncheckpointinhibitoren ist deswegen sehr wahrscheinlich. Für eine detailliertere Darstellung des TAP-Scores sei auf die aktuelle Literatur verwiesen [15].

#### Tumor Proportion Score

Der Tumor Proportion Score (TPS) ist insbesondere für Plattenepithelkarzinome des Ösophagus etabliert, die eine häufigere PD-L1-Expression in Tumorzellen zeigen als Adenokarzinome [2]. Der TPS bezeichnet den Prozentsatz der vitalen Tumorzellen mit partieller oder kompletter membranöser PD-L1-Färbung, unabhängig von der Intensität. Die Bewertung erfolgt analog zur Lungenkarzinomdiagnostik.

Die **Scoreauswahl**

Scoreauswahl

richtet sich nach der geplanten Therapie und der Tumorentität [6, 7]. In der Praxis werden häufig mehrere Scores parallel angegeben, um alle Optionen offenzuhalten. Bei den Adenokarzinomen ist für Pembrolizumab ein CPS ≥ 10 erforderlich, für Nivolumab ein CPS ≥ 5. Die Tislelizumabzulassung durch die European Medicines Agency (EMA) basiert auf einem TAP-Score ≥ 5 %. Die PD-L1-Expression ist in Adenokarzinomen meist auf Immunzellen beschränkt, eine Tumorzellexpression ist seltener als in Plattenepithelkarzinomen. Bei diesen ist für Nivolumab ein TPS ≥ 1 % erforderlich, für Pembrolizumab ein CPS ≥ 10. Tislelizumab ist wie bei Adenokarzinomen bei vorliegendem TAP-Score ≥ 5 % zugelassen. In der kürzlich publizierten **MATTERHORN-Studie**

MATTERHORN-Studie

wird ein TAP-Score ≥ 1 % als möglicher Stratifikationsparameter für den ergänzenden perioperativen neoadjuvanten Einsatz von Durvalumab zur FLOT-Standardtherapie in Magen- und Übergangsadenokarzinomen untersucht [14].

##### Merke

Die Bestimmung des PD-L1 Status erfolgt mittels Angabe des TPS (Tumor Positivity Score), des CPS (Combined Positivity Score) und fakultativ des TAP (Tumor Area Positivity) Scores.

### Mikrosatelliteninstabilität/Mismatch-repair-Defizienz

Die Testung auf MSI bzw. MMR-Defizienz (dMMR) besitzt neben der Identifikation hereditärer Tumorsyndrome wie das Lynch-Syndrom ebenfalls eine hohe **prädiktive Relevanz**

prädiktive Relevanz

für den Einsatz von Immuncheckpointinhibitoren. Karzinome des oberen Gastrointestinaltrakts vom MSI-high-Typ sind häufig durch somatische Ereignisse wie die **Promotorhypermethylierung**

Promotorhypermethylierung

von *MLH1* verursacht. Etwa 8–11 % der Magenkarzinome fallen in die MSI/dMMR-Subgruppe [16]. Diese Tumoren sind häufig im älteren Patientenkollektiv zu finden, zeigen oft eine intestinale Histologie und sind vorwiegend im Korpus und Antrum lokalisiert. Sie sind durch eine Vielzahl von Insertions- und Deletionsmutationen, eine ausgeprägte lymphozytäre Infiltration sowie eine hohe Expression von PD-L1 charakterisiert.

Die immunhistochemische Untersuchung auf die **4 MMR-Proteine**

4 MMR-Proteine

(MLH1, PMS2, MSH2, MSH6) ist das empfohlene Standardverfahren in der Routinediagnostik [17]. Ein vollständiger Verlust der nukleären Expression eines oder mehrerer dieser Proteine in den Tumorzellen, bei erhaltener Expression in internen Kontrollzellen (Lymphozyten, Stromazellen, nichtneoplastisches Epithel), ist für eine MMR-Defizienz beweisend. Aufgrund der **Heterodimerstruktur**

Heterodimerstruktur

der MMR-Proteine führen Alterationen in MLH1 meist auch zum Verlust von PMS2, Alterationen in MSH2 zum Verlust von MSH6. Isolierte Verluste von PMS2 oder MSH6 sind selten, aber möglich und bedürfen einer weiteren Abklärung mittels molekularer Methoden.

Als Goldstandard für die direkte Bestimmung der MSI gilt die Analyse von Mikrosatellitenmarkern mittels **Polymerasekettenreaktion**

Polymerasekettenreaktion

(PCR). Hierbei werden Panels aus Mono- und Dinukleotid-Repeats (z. B. BAT-25, BAT-26, NR-21, NR-24, NR-27) untersucht. Eine Instabilität in ≥ 2 Markern definiert den MSI-high-Status. Die PCR-basierte Methode ist besonders dann indiziert, wenn die IHC uneindeutig ist (z. B. durch bekannte Artefakte wie eine granuläre nukleäre Anfärbung bei der MLH-1-Färbung, die nicht als erhaltene Expression gewertet werden sollte) oder atypische Expressionsmuster vorliegen. Die Konkordanz zwischen MMR- und MSI-Status liegt jedoch bei fast 100 % [18]. Alternativ oder ergänzend kann auch eine Next-Generation-Sequencing(NGS)-basierte Analyse erfolgen, die neben MSI auch die Tumormutationslast und weitere molekulare Alterationen erfassen kann.

Sowohl im lokalisierten als auch im metastasierten Stadium profitieren PatientInnen mit MSI/dMMR-Tumoren signifikant von einer **Immuncheckpointblockade**

Immuncheckpointblockade

(z. B. Pembrolizumab, Nivolumab), unabhängig von der PD-L1-Expression. Die Ansprechrate liegt deutlich über der von MSS/pMMR-Tumoren, was in mehreren Studien bestätigt wurde [8]. Auch im kurativen Setting zeigen PatientInnen mit MSI/dMMR-Tumoren ein besseres Überleben, profitieren jedoch weniger von einer adjuvanten Chemotherapie, sodass in frühen Stadien eine Operation allein ausreichend sein kann.

### Claudin (CLDN) 18.2

Claudine gehören zu einer Familie von Tight-junction-Proteinen, die den **parazellulären Transport**

parazellulären Transport

und die Zellpolarität regulieren. Das Protein CLDN18 existiert in 2 Isoformen: CLDN18.1, das vorwiegend in der Lunge, und CLDN18.2, das physiologisch ausschließlich in der Magenschleimhaut exprimiert wird. Im Rahmen der malignen Transformation bleibt CLDN18.2 erhalten, wird jedoch durch den Verlust der epithelialen Polarität für Antikörper zugänglich. Dadurch wird CLDN18.2 zu einem attraktiven Ziel für antikörperbasierte Therapien, da es in anderen Geweben kaum vorkommt und somit eine **hohe Tumorspezifität**

hohe Tumorspezifität

aufweist. In Magenkarzinomen findet sich eine moderate bis starke membranöse Expression von CLDN18.2 in etwa 30 % der Fälle, unabhängig vom histologischen Tumortyp [19].

**Abb. 3:**
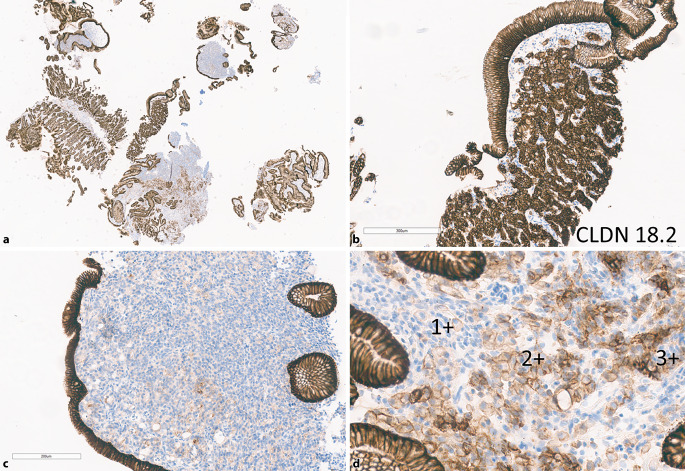
Beispiel einer immunhistochemischen CLDN18.2-Färbung mit heterogenem Expressionsmuster. **a** Übersicht. **b** Areal mit einer Reaktivität von ausschließlich 3+ in den Tumorzellen. **c** Areal mit einer Reaktivität von 0/1+. **d** Areal mit einem Reaktivitätslevel von 0 bis 3+ in den Tumorzellen. Der Fall wurde von 3 PathologInnen übereinstimmend gänzlich als „negativ“ mit 50 % 2+/3+ gewertet. Zu beachten ist auch die interne Positivkontrolle des normalen Magenepithels

Die Bestimmung von CLDN18.2 erfolgt mittels IHC mithilfe eines geeigneten Assays [20]. Verschiedene Testplattformen und Antikörper scheinen gleichwertig zu sein. Als positiv gilt eine moderate bis starke (2+/3+) membranöse Färbung (komplett, basolateral oder lateral) in mindestens 75 % der Tumorzellen. Es wird empfohlen, die Bewertung analog der bereits erwähnten HER2-Bestimmung vorzunehmen [21]. Als **interne Positivkontrolle**

interne Positivkontrolle

dient die normale Magenschleimhaut, die eine starke membranöse CLDN18.2-Expression zeigt (Abb. 3). Bereiche mit intestinaler Metaplasie können ebenfalls als interne Kontrolle genutzt werden, wobei die Expression hier schwächer ausfällt. Lymphozyten, glatte Muskulatur und Gefäße färben sich nicht an. Bei der Untersuchung von Metastasen, in denen keine interne Positivkontrolle vorhanden ist, sollte Magenschleimhaut als externe Kontrolle mitgeführt werden [21]. Im Befundbericht empfiehlt es sich zudem, die Anzahl der positiven Zellen mit 2+ bzw. 3+ und ggf. die Wertung als „positiv“ vs. „negativ“ als Zusatz anzugeben und nicht nur die dichotomische Unterscheidung anhand des derzeitig gültigen Schwellenwerts, um im derzeitigen frühen Stadium des Erkenntnisgewinns mit diesem Biomarker keine Primärdaten zu verlieren [21].

Die Zulassung von Zolbetuximab für CLDN18.2-positive, HER2-negative, fortgeschrittene oder metastasierte Magen- und GEJ-Adenokarzinome basiert auf 2 Phase-III-Studien (SPOTLIGHT, GLOW), in denen die Kombination von Zolbetuximab mit Chemotherapie das Gesamtüberleben im Vergleich zu alleiniger Chemotherapie signifikant verbesserte. Zukünftig werden weitere **Anti-CLDN18.2-Therapien**

Anti-CLDN18.2-Therapien,

darunter bispezifische Antikörper, Antikörper-Wirkstoff-Konjugate (ADC) und chimäre Antigenrezeptoren exprimierende T‑Zellen (CAR-T-Zellen) in klinischen Studien geprüft. Es ist in diesem Kontext zu erwarten, dass klinisch relevante Cut-off-Werte für die CLDN18.2-Expression therapieabhängig vom derzeitigen 75 %-Wert abweichen werden.

### Ausblick – andere Zielmoleküle

Das FGFR2-Protein ist ein Tyrosinkinaserezeptor, der durch Bindung von FGF zahlreiche Signalwege aktiviert, die Zellproliferation, Migration und Überleben fördern. Die Isoform FGFR2b wird physiologisch vor allem im Magenepithel exprimiert, wobei es während der Tumorgenese zu einer Überexpression oder Amplifikation kommen kann. Während die *FGFR2*-Gen-Amplifikation im Magenkarzinom relativ selten ist (etwa 3–9 %), findet sich immunhistochemisch eine Überexpression von FGFR2b-Protein in bis zu 30–50 % der Fälle [17]. In der randomisierten Phase-II-Studie FIGHT wurde der FGFR2b-Inhibitor Bemarituzumab in Kombination mit Chemotherapie erfolgreich bei FGFR2b-überexprimierenden, HER2-negativen Magen- und GEJ-Adenokarzinomen eingesetzt [8], wobei die therapeutische Wirksamkeit von FGFR2b-Inhibitoren – anders als bei HER2 – weniger von der Genamplifikation als vielmehr von der Proteinüberexpression abzuhängen scheint. Die Bestimmung der FGFR2b-Expression (membranöse Färbung) mittels IHC rückt deshalb derzeit als prädiktiver Biomarker in den Fokus [22].

Fusionen unter Beteiligung der Gene, die die Familie der **neurotropen Tyrosinrezeptorkinasen**

neurotropen Tyrosinrezeptorkinasen

(NTRK) kodieren, sind beim Magenkarzinom seltene, aber therapeutisch prinzipiell relevante Alterationen. Sie führen zur konstitutiven Aktivierung von TRK-Proteinen, was eine onkogene Signaltransduktion begünstigt. Die Prävalenz von **NTRK-Fusionen**

NTRK-Fusionen

liegt bei Magen- und gastroösophagealen Adenokarzinomen allerdings deutlich unter 1 %, sie können jedoch insbesondere bei jüngeren Patienten oder in MSI-H/dMMR-Tumoren auftreten. Ihr Nachweis eröffnet die Option einer Therapie mit TRK-Inhibitoren (z. B. Larotrectinib, Entrectinib), die tumoragnostisch zugelassen sind [23].

## Präanalytische und analytische Aspekte

Ein zentrales Problem der Biomarkertestungen von Karzinomen im oberen Gastrointestinaltrakt, speziell von Adenokarzinomen, ist die teils ausgeprägte räumliche und zeitliche **Heterogenität**

Heterogenität

der Expression der Zielmoleküle [24]. In bis zu 75 % der Fälle kann z. B. eine Heterogenität der HER2-Expression vorliegen, sowohl zwischen verschiedenen Tumorarealen als auch zwischen Primärtumor und Metastasen. Zudem kann unter Therapie ein Verlust der HER2-Expression auftreten, was insbesondere bei geplanter Zweitlinientherapie (z. B. Trastuzumab-Deruxtecan) eine erneute Testung erforderlich macht. Ähnliche Daten liegen auch für PD-L1 und  CLDN 18.2 vor [6, 7]. Im Gegensatz dazu zeigt der MMR/MSI-Status nur in sehr seltenen Ausnahmefällen eine räumliche oder zeitliche Heterogenität [25].

Um dieser Heterogenität Rechnung zu tragen, sollten in der Biopsiediagnostik mindestens 8, davon mindestens 5 tumortragende Biopsien entnommen werden, die für die Testung verwendet werden können. Für PD-L1 und HER2 wurde zudem eine Mindestanzahl an zusammenliegenden Tumorzellen vorgeschlagen, die für eine **zuverlässige Biomarkerdiagnostik**

zuverlässige Biomarkerdiagnostik

vorliegen sollte.

### Merke

Für eine zuverlässige Biomarkerdiagnostik an Biopsien sollten mindestens 5 tumortragende Biopsien vorliegen.

Bei HER2 wird diese Mindestmenge mit 50 vitalen Tumorzellen angegeben. Diese Zahl orientiert sich an der für eventuell nötige ISH-Untersuchungen üblicherweise geforderten Menge von Tumorzellen. Bei PD-L1 wird in Anlehnung an die Lungenkarzinomdiagnostik [26] eine Menge von 100 vitalen Tumorzellen empfohlen. Für CLDN18.2 wird empfohlen, sich an HER2 zu orientieren, wobei in der hier relevanten Publikation explizit angegeben wird, dass 100 Zellen besser sind als 50 [21]. In jedem Fall sollte ein Block mit ausreichender Tumorzellzahl untersucht werden, bei morphologischer Heterogenität oder zu wenigen Tumorzellen mehrere Blöcke. Ein Kommentar über eingeschränkte **Repräsentativität**

Repräsentativität

im pathologischen Befundbericht bei Unterschreiten der empfohlenen Mindestanzahl von Biopsien und Tumorgehalt ist sinnvoll. Aufgrund der in der Literatur beschriebenen Diskordanz zwischen Primärtumor und Metastasen sowie vor und nach Chemotherapie ist zudem eine **erneute Testung**

erneute Testung

bei Progression oder Metastasierung sinnvoll, auch wenn sie derzeit nicht explizit in Leitlinien gefordert ist. Aufgrund eines möglichen Antigenverlustes bei der Untersuchung von älteren Paraffinblöcken sollte das jüngste verfügbare Material untersucht werden, sofern dies mit den anderen bereits genannten Qualitätskriterien vereinbar ist. Eine Festlegung, welches Material zu verwenden ist, kann auch z. B. im Rahmen eines Tumorboards diskutiert werden, wobei die Qualitätskriterien vor allem hinsichtlich des Tumorgehalts von Seiten der Pathologie überprüft werden müssen bzw. bereits im Befund angegeben sein sollten. Im Zweifelsfall sollten die Testungen an mehreren Blöcken oder Gewebearten (d. h. Biopsie, Resektat, Metastasen) durchgeführt werden [12].

Neben technischen und biologischen Herausforderungen ist auch die **untersucherbedingte Variabilität**

untersucherbedingte Variabilität

zu beachten. Gerade bei grenzwertigen Fällen sollte ein:e zweite:r Untersucher:in hinzugezogen werden, was insbesondere bei CLDN18.2 für Fälle mit 60–80 % Zellen der Kategorie 2+/3+ empfohlen wird, da hier im Gegensatz z. B. zu HER2 keine weitere alternative Untersuchungsmethoden wie eine In situ-Hybridisierung zur Verfügung steht [21]. Zu erwarten ist zudem eine Erleichterung der Biomarkerbewertung durch den Einsatz von **digitalen Analysemethoden**

digitalen Analysemethoden.

Studien zeigten bereits, dass u. a. die Vorhersage einer MSI bei Magenkarzinomen mittels auf künstlicher Intelligenz (KI) basierenden Methoden am gescannten Hämatoxylin-Eosin(HE)-Schnitt mit sehr guten Ergebnissen möglich ist [27]. Auch für PD-L1 und HER2 wurden KI-basierte Auswertemethoden bereits erfolgreich angewandt, wenn auch an gastrointestinalen Karzinomen bislang in nur begrenztem Umfang im Vergleich zu anderen Tumorarten. Aufgrund der derzeitigen rasanten Entwicklung von prädiktiven computergestützten Ansätzen in der (digitalen) Pathologie ist jedoch davon auszugehen, dass solche Methoden auch in der Biomarkeranalytik für Karzinome des oberen Gastrointestinaltrakts Einzug halten werden [28].

## Fazit für die Praxis

- Zielgerichtete Therapien mit biomarkerbasierten Ansätzen bieten wesentliche Überlebensvorteile bei Tumoren des oberen Gastrointestinaltrakts.
- Derzeit umfasst das prädiktive Biomarkerpanel für gastroösophageale Adenokarzinome HER2, „programmed death ligand 1“ (PD-L1; Tumor Proportion Score [TPS], Combined Positivity Score [CPS], neu auch Tumor Area Proportion [TAP] Score), Mikrosatelliteninstabilität (MSI)/ Mismatch-repair-Defizienz (dMMR), Claudin 18.2 (CLDN18.2) und für ösophageale Plattenepithelkarzinome PD-L1 (TPS, CPS, neu auch TAP Score).
- Bei der Biomarkerdiagnostik ist vor allem aufgrund der zu beobachtenden biologischen Heterogenität der Markerexpression auf die Einhaltung strenger Qualitätsrichtinien, insbesondere was die Quantität der zu untersuchenden Tumorzellen angeht, zu achten.
- Unter diesem Aspekt wird im Verlauf einer Tumorerkrankung eine repetitive Biomarkerdiagnostik empfohlen.

## CME-Fragebogen

Welche/r Biomarker sollten routinemäßig bei (fortgeschrittenen oder metastasierten) Magenkarzinomen bestimmt werden?

HER2

PD-L1

MSI/MMR-Status

Claudin 18.2

Alle der genannten

Welche Methoden werden üblicherweise bei der Bestimmung des HER2-Status in Magenkarzinomen angewandt?

Immunhistochemie und PCR

Next Generation Sequencing und In-situ-Hybridisierung

Immunhistochemie und In-situ-Hybridisierung

Immunhistochemie und Next Generation Sequencing

In-situ-Hybridisierung und PCR

Ein 62-jähriger Patient mit einem metastasierten Plattenepithelkarzinom des Ösophagus wird für eine Immuntherapie in Betracht gezogen. Welcher Aussage ist am relevantesten?

Der klinisch relevante Schwellenwert für die PD-L1-Expression liegt therapieunabhängig bei einem Tumor Proportion Score (TPS) von 10 %.

Die Bestimmung des HER2-Status ist bei metastasierten Tumoren klinisch am relevantesten.

Bei Plattenepithelkarzinomen des Ösophagus ist der Combined Positivity Score (CPS) der relevante Score für die Therapieentscheidung.

Mutationen im FGFR2b-Gen sind aufgrund aktueller klinischer Daten vielversprechende therapeutisches Targets.

Eine Mikrostatelliteninstabilität ist in ca. 10 % der Fälle zu beobachten.

Was wird für den CPS (Combined Positivity Score) bei PD-L1 bestimmt?

PD-L1-positive Tumorzellen und mononukleäre Immunzellen (Lymphozyten, Makrophagen) im Verhältnis zur Gesamtzahl vitaler Tumorzellen

Fläche von PD-L1-positiven Tumorzellen und aller Immunzellen (Lymphozyten, Makrophagen, Granulozyten) im Verhältnis zur Tumorfläche

PD-L1-positive Immunzellen (Lymphozyten, Makrophagen, Granulozyten) innerhalb der Tumorfläche

PD-L1-positive Tumorzellen im Verhältnis zur Gesamtzahl vitaler Tumorzellen

PD-L1-positive Tumorzellen und mononukleäre Immunzellen (Lymphozyten, Makrophagen) im Verhältnis zur Tumorfläche

Eine 58-jährige Patientin mit einem metastasierten Adenokarzinom des Magens und einem PD-L1-positiven Tumorstatus wird für eine Immuntherapie beurteilt. Welcher Tumortyp zeigt am häufigsten ein hohes PD-L1-Expressionsniveau, was die Therapieentscheidung beeinflusst?

Plattenepithelkarzinom des Ösophagus

Mikrosatelliteninstabiles Magenadenokarzinom

Magenkarzinom vom wenig kohäsiven Typ

Ösophageales Adenokarzinom, intestinaler Typ

Muzinöses Adenokarzinom des Magens

Welches Phänomen ist bei der HER2-Testung diagnostisch und klinisch am relevantesten?

Diskrepanz zwischen Immunhistochemie und In-situ-Hybridisierung

Räumliche und zeitliche Heterogenität

Unspezifisches granuläre Färbemuster

Interobserver-Variabilität

Verlust der Färbeintensität bei alten FFPE-Blöcken

Welches Färbemuster wird bei der Claudin (CLDN)-18.2-Färbung als spezifisch angesehen?

Nukleär

Zytoplasmatisch

Membranär

Granulär

Expressionsverlust

Wie hoch ist die Übereinstimmung zwischen molekularpathologischer Bestimmung der Mikrosatelliteninstabilität und der immunhistochemischen Bestimmung auf Mismatch-repair-Defizienz in gastrointestinalen Tumoren

60 %

70 %

80 %

90 %

Fast 100 %

Welche Funktion haben Claudine?

Signaltransduktion

Tyrosinkinasen

DNA-Reparatur

Tight-junction-Proteine

Apoptose

Was dient bei der Claudin (CLDN)-18.2-Bestimmung als interne Positivkontrolle?

Lymphozyten

Makrophagen

Normales Magenepithel

Cajal-Zellen

Neuroendokrine Zellen
